# A Novel and Potentially Multifaceted Dehydroascorbate Reductase Increasing the Antioxidant Systems is Induced by Beauvericin in Tomato

**DOI:** 10.3390/antiox9050435

**Published:** 2020-05-16

**Authors:** Martina Loi, Silvana De Leonardis, Giuseppina Mulè, Antonio F. Logrieco, Costantino Paciolla

**Affiliations:** 1 Institute of Sciences of Food Production, National Research Council, Via G. Amendola 122/0, 70126 Bari, Italy; martina.loi@ispa.cnr.it (M.L.); giuseppina.mule@ispa.cnr.it (G.M.); antonio.logrieco@ispa.cnr.it (A.F.L.); 2 Department of Biology, University of Bari “Aldo Moro”, Via E. Orabona 4, 70125 Bari, Italy; silvana.deleonardis@uniba.it

**Keywords:** beauvericin, dehydroascorbate reductase, tomato, 1,7-sedoheptulose bisphosphatase, RNA-binding protein, antioxidants, ascorbate

## Abstract

Dehydroascorbate reductases (DHARs) are important enzymes that reconvert the dehydroascorbic acid (DHA) into ascorbic acid (ASC). They are involved in the plant response to oxidative stress, such as that induced by the mycotoxin beauvericin (BEA). Tomato plants were treated with 50 µM of BEA; the main antioxidant compounds and enzymes were evaluated. DHARs were analyzed in the presence of different electron donors by native and denaturing electrophoresis as well as by western blot and mass spectrometry to identify a novel induced protein with DHAR activity. Kinetic parameters for dehydroascorbate (DHA) and glutathione (GSH) were also determined. The novel DHAR was induced after BEA treatment. It was GSH-dependent and possessed lower affinity to DHA and GSH than the classical DHARs. Interestingly, the mass spectrometry analysis of the main band appearing on sodium dodecyl sulphate polyacrylamide gel electrophoresis (SDS-PAGE) revealed a chloroplast sedoheptulose 1,7-bisphosphatase, a key enzyme of the Calvin cycle, and a chloroplast mRNA-binding protein, suggesting that the DHA reducing capacity could be a side activity or the novel DHAR could be part of a protein complex. These results shed new light on the ascorbate-glutathione regulation network under oxidative stress and may represent a new way to increase the plant antioxidant defense system, plant nutraceutical value, and the health benefits of plant consumption.

## 1. Introduction

**Figure 1:**
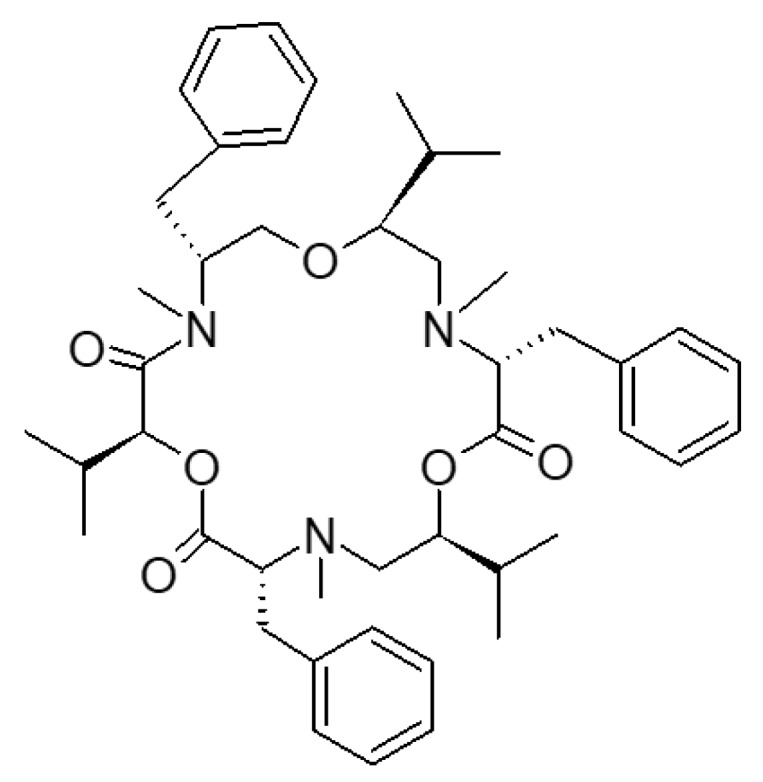
Beauvericin chemical structure.

Beauvericin (BEA) is a mycotoxin produced by several *Fusarium* spp., such as *F. proliferatum*, *F. subglutinans*, *F. verticillioides*, and *F. oxysporum*. It is mostly found on cereal grains and their by-products [1]. Since 2008, BEA was addressed as an emerging mycotoxin, along with the structurally related enniatins and the other *Fusarium* toxins moniliformin and fusaproliferin [2]. From a chemical point of view, BEA is a hexa depsipeptide (Figure 1), produced by a multifunctional enzyme enniatin synthetase, which catalyzes both amino acid condensation and cyclicization steps [3]. 

BEA possesses a wide range of biological activities. It is an ionophore, it induces oxidative stress, acts as an antimicrobial, and as an enzyme inhibitor. The LD_50_ for acute toxicity in mice was estimated to be 100 mg/kg; however, toxicity in humans has not been reported yet [4]. Cytotoxicity is mostly due to the selective ionophore (channel-forming) activity on biological membranes, which allows a flux of cations, particularly Ca^2+^, into the cell [5]. 

Due to its cytotoxicity, it has been employed in the treatment of malignant cells [6], as an antibacterial [7], antifungal [8], insecticidal, and nematocidal molecule [9]. The use of BEA for crop management has to be carefully evaluated because it can induce cell death and alteration of the antioxidant defense system in plants, such as the ascorbate-glutathione pathway [10,11]. This defense system is composed by enzymes such as the ascorbate peroxidase (APX, EC 1.11.1.11), the monodehydroascorbate reductase (MDHAR, EC 1.6.5.4), the dehydroascorbate reductase (DHAR, EC 1.8.5.1), and the glutathione reductase (GR, EC 1.6.4.2), and compounds, such as ascorbate (ASC), dehydroascorbate (DHA), reduced (GSH) and oxidized (GSSG) glutathione. In this pathway, DHAR uses GSH to reduce DHA generated from the oxidation of ASC, thereby regenerating it [12]. 

This underlines that this enzyme plays a critical role in the ASC-GSH recycling reaction in higher plants [13]. DHAR activity has been identified in cytosol, chloroplasts, mitochondria, and peroxisomes [14], the latter three organelles being the compartments where the electron transport chains and photochemical reactions take place and reactive oxygen species (ROS) are generated the most [15]. So far, three different genes coding for DHAR have been found in *Arabidopsis*; they were localized in cytosol (*DHAR2*), peroxisomes (*DHAR1*), and in chloroplasts (*DHAR3*) [16]. In the cell, the excessive ROS generation causes oxidative damage. Nevertheless, they also have physiological functions, because the ROS act as signal molecules and participate in the stress response signal to induce adaptation [15]. Thus, the antioxidant systems have to re-establish and maintain a physiological ROS balance.

Antioxidant compounds possess a wide range of health benefits [17], and plants are the most important source of many of them, such as ASC [18], polyphenols [19], tocopherols, carotenoids, xanthophylls [20], and many others [21]. They are critical to counteract the ROS, which are physiologically generated in plant metabolism and extensively produced in response to biotic or abiotic stress. Particularly, the enzymatic antioxidant components of the ASC-GSH pathway participate in ROS-scavenging [15]. In fact, many of these enzymes, such as DHAR, can be regulated during extreme environmental conditions, nutrient deficiency, or in presence of high salinity to confer high resistance and improved photosynthetic efficiency [22,23]. Thus, DHAR regulates the cell ASC redox state, which is responsible for the cell defense response and tolerance to several biotic and abiotic stresses [13,24,25]. Additionally, it has been demonstrated that overexpression of DHAR, but not MDHAR, confers stress tolerance in transgenic tobacco [26].

However, additional studies are needed to identify the prominent physiological role(s) of DHAR in the contribution to ascorbate recycling and stress response in horticultural crops of economic relevance. In a previous work, the effect of *Fusarium* mycotoxins treatment on tomato young plants was evaluated [27]. The treatment with T-2 toxin caused a marked wilting and oxidative stress, while no significant alterations were visible for BEA treated plants, underling in these latter the capacity of the tomato plants to counteract BEA induced oxidative stress. 

Therefore, the aim of this work was to provide a more in-depth investigation of the antioxidant defense system of tomato plants that allow them to cope with BEA-induced oxidative stress, with particular regards to the ASC-regenerating enzyme DHAR.

## 2. Materials and Methods 

### 2.1. Chemicals

All reagents used in this study were of the highest grade available. They were purchased from Sigma-Aldrich (Milan, Italy) and used without further purification. BEA standard was dissolved in methanol to obtain a 1.28 mM stock solution. Ultrapure water was produced by a Milli-Q system (Millipore, Bedford, MA, USA).

### 2.2. Plant Material

Tomato seeds (*Lycopersicon esculentum* L. cv. Marmande) were purchased from a local market and germinated under a white fluorescent light (12 h photoperiod) at 23 ± 1 °C and with 55 ± 2% of relative humidity. After 12 days, plants were cut at the collar level and the shoots were incubated in H_2_O containing 50 μM of BEA or an equivalent amount of methanol (control). After 12, 24, and 36 h, the plants were washed with distilled water and analyzed.

### 2.3. Determination of Ascorbate and Glutathione Pools

Five grams of shoots were grounded in a porcelain mortar with 10 mL of cold solution containing 5% metaphosphoric acid. Then, ASC, DHA, GSH and oxidized glutathione (GSSG) were determined spectrophotometrically as reported by Paciolla and colleagues [27].

### 2.4. Proteins Extraction and Quantification

Shoots were homogenized at 4 °C in 50 mM Tris-HCl pH 7.8 containing 0.3 mM mannitol, 1 mM EDTA, and 0.05% (*w*/*v*) cysteine. To obtain the cytosolic fraction, the homogenate was centrifuged at 1000× *g* for 5 min and then the supernatant was re-centrifuged for 20 min at 25,000× *g*. Finally, the cytosolic fraction was desalted by dialysis against 50 mM Tris-HCl, pH 7.8. This desalted fraction was used for enzyme activity measurements and for the electrophoretic analyses. The protein content was quantified with a Protein Assay kit from Bio-Rad (Hercules, CA, USA) with bovine serum albumin as the standard.

### 2.5. Enzyme Activity Measurements

DHAR activity was measured spectrophotometrically by monitoring ASC production at 265 nm (extinction coefficient 14 mM^−1^ cm^−1^). A control experiment was also performed to assess ASC non-enzymatic reduction. The reaction mixture contained 50 µg of protein, 1 mM DHA, 2 mM GSH and 100 mM phosphate buffer, pH 6.3. The activities of other enzymes, namely APX, MDHAR, and GR were analyzed as reported in [27].

To evaluate the GSH-dependence of DHAR activity, some plants were simultaneously incubated with BEA 50 µM and buthionine-[*S*,*R*]-sulfoximine (BSO), an inhibitor of GSH synthesis, at 100 µM. Proteins were extracted as reported in Section 2.4. and loaded on native polyacrylamide gel electrophoresis (PAGE).

### 2.6. Electrophoretic Analyses

#### 2.6.1. Native-Polyacrylamide Gel Electrophoresis

Native-PAGE was performed on PAGE (4.3% T; 7.3% C) with a running buffer consisting of 4 mM Tris-HCl pH 8.3 and 38 mM glycine. The extracted proteins were loaded in duplicate on the same gel. Fourty μg of total proteins were loaded in each lane. After the electrophoretic run, the gel was divided into two parts: the first one was used for the activity staining, while the second one was transferred to the polyvinylidene difluoride (PVDF) membranes (Sigma-Aldrich, Milan, Italy) for the western blot analysis (see Section 2.7).

For DHAR activity staining, the gel was incubated for 15 min in 0.1 M Na-phosphate buffer pH 6.2, containing 4 mM GSH and 2 mM DHA. To further test GSH-dependence of the enzyme, other electron donors were tested alternatively to GSH in the native-PAGE, namely NADPH, alpha-lipoic acid or DL-lipoamide at the same concentration. Finally, gels were incubated for 15 min in the dark with a solution of 0.125 N HCl containing 0.1% (*w*/*v*) potassium ferricyanide and 0.1% (*w*/*v*) ferric chloride. With this coloration, DHARs appear as dark blue bands on a light blue background, the latter due to the non-enzymatic ASC formed due to the reaction between DHA and GSH, NADPH, alpha-lipoic acid, or dl-lipoamide.

#### 2.6.2. Sodium Dodecyl Sulphate Polyacrylamide Gel Electrophoresis

The novel band was excised from the native gel with a scalpel and grounded in a porcelain mortar with a solution containing Tris 0.016 M, Glycine 0.152 M, pH 8.3 in a 1:3 weight/volume ratio. The homogenate was centrifuged at 14,000× *g* for 20 min at 4 °C. The protein content of the obtained supernatant was assayed with the Bio-Rad kit (see Section 2.4).

For protein identification by the estimation of the molecular weight and mass spectrometry analysis, 15 μg of total protein were loaded on sodium dodecyl sulphate (SDS) PAGE (12% T, 3% C), performed according to Laemmli [28]. The protein content was assayed with the Bio-Rad kit (see Section 2.4).

Electrophoretic separation was performed in a Mini Protean System (Bio-Rad, Segrate, Italy) filled with running buffer composed of 25 mM Tris and 1.9 M glycine. The run was performed at 100 mV for 15 min, then at 150 mV for 1.5 h. After the run, the gel was washed twice with distilled water, fixed with 40% methanol, 10% acetic acid, and 50% H_2_O for 30 min and then stained with 50% methanol, 50% H_2_O, and 0.8% *w*/*v* Coomassie R250 for 1 h. Then, the gel was destained with 50% H_2_O, 40% methanol, and 10% acetic acid for 6 h. Images were acquired with a digital photographic apparatus. 

### 2.7. Western Blot Analysis

Immunoblots were performed using PVDF membranes, using polyclonal DHAR1 rabbit IgG (catalog number AS11 1746, Agrisera, Sweden) at 1:5000 dilution according to the manufacturer’s’ instructions (www.agrisera.com). Positive signals were visualized after 2 h of incubation at room temperature with 3’-diaminobenzidine, used as substrate for horseradish peroxidase-conjugated goat anti-rabbit IgG at 1:10,000 dilution.

### 2.8. Protein Identification by Mass Spectrometry 

The band from the SDS-PAGE was excised, cut into small pieces and in-gel digested: trypsin was chosen as proteolytic enzyme. Protein digestion was performed according to the manufacturer’s instructions. The tryptic digest was analyzed by LC-nano-ESI-ion trap analysis (LC-MS/MS) (LC/MCD-Trap-XCT-Ultra, Agilent-Technologies, Palo Alto, CA). Peptide separation was performed using a Zorbax 300SB reverse phase C18 column (150 mm × 0.075 mm, 3.5 µm). The following conditions were used for the analytical separation: 5–70% acetonitrile gradient in 0.1% formic acid over 55 min, with a flow rate of 0.3 µL/min. Spectra acquisition was performed in Data-dependent scan modality and analyzed using Mascot Search (http://www.matrixscience.com/) and Spectrum Mill (Agilent Technologies, Palo Alto, CA, USA) software. The protein search was performed against a customized database (UniProt DB, available online) containing approximately 36,880 entries referred to *Lycopersicon esculentum* species.

### 2.9. Kinetic Measurements

Kinetic parameters (*K*_M_ and *V*_max_) were determined using 1.12 µg of eluted protein (see Section 2.6.2) and increasing amount of DHA (0.3, 0.5, 0.7, 1.0, 1.3, 1.5, 1.6, 1.7, 1.8, 1.9, 2.0, 2.3, 2.4, 2.5, 3.0, 4.0, 5.0 mM) with saturating concentration of GSH (2 mM) or GSH (0.5, 1.0, 1.5, 2.0, 2.5, 3.0, 4.0, 6.0, 9.0, 12.0, 15.0 mM) with saturating concentration of DHA (1 mM). The DHA and GSH conversion was calculated as reported in Section 2.5. Data were fitted using GraphPad Prism version 8.0.0 for Windows, GraphPad Software, San Diego, CA, USA, www.graphpad.com.

### 2.10. Statistical Analyses

Data presented are the mean of five different experiments ± standard deviation (SD). Samples were compared by Student’s *t* test. Differences were considered significant for *p* < 0.05 and highly significant for *p* < 0.01.

## 3. Results

### 3.1. Determination of Ascorbate and Glutathione Pools

**Figure 2:**
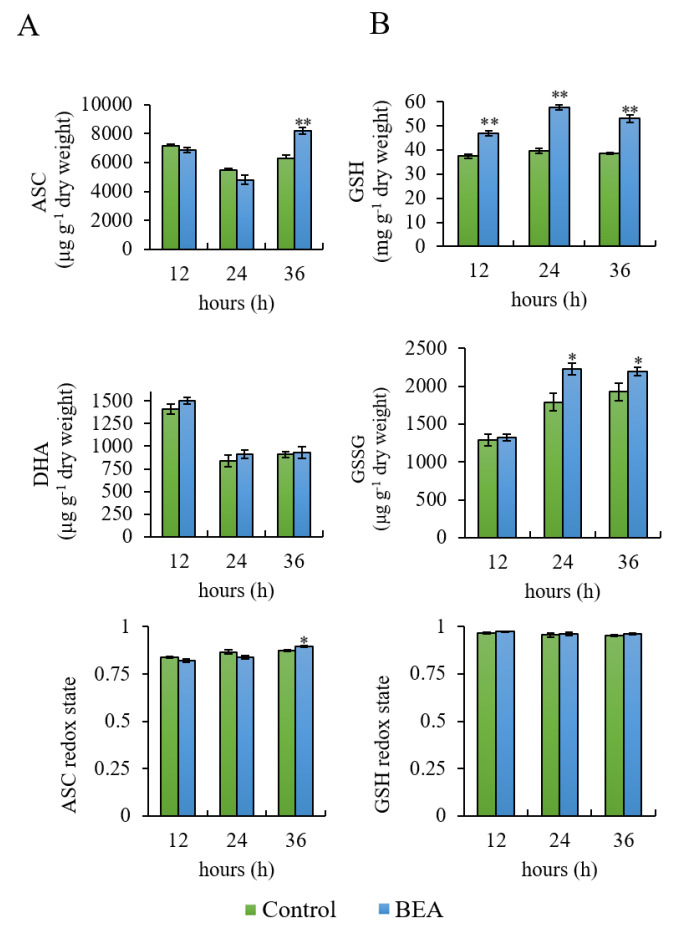
Ascorbate (ASC) (Panel **A**) and glutathione (GSH) (Panel **B**) pools in control and beauvericin (BEA) treated sample during 36 h of assay. Asterisks indicate values significantly different from the control at each time point, according to the Student’s *t* test with *p* < 0.05 (*) and *p* < 0.01 (**).

ASC trend in the control and BEA treated sample was the same and consisted of a slight decrease after 24 h and an increase after 36 h (Figure 2, Panel A). Differences between samples were statistically significant after 36 h (*p* < 0.01). As regards DHA content, no differences between samples were registered throughout the assay. The ASC redox state (ASC/ASC + DHA) was only slightly higher after 36 h (*p* < 0.05) (Figure 2, Panel A).

As regards GSH pool, a significant increase in both GSH (*p* < 0.01) and GSSG (*p* < 0.05) was registered at almost each time point (Figure 2, Panel B). Nevertheless, the GSH redox state (GSH/GSH + GSSG) was not altered (Figure 2, Panel B).

### 3.2. Enzyme Activity Measurements

**Figure 3:**
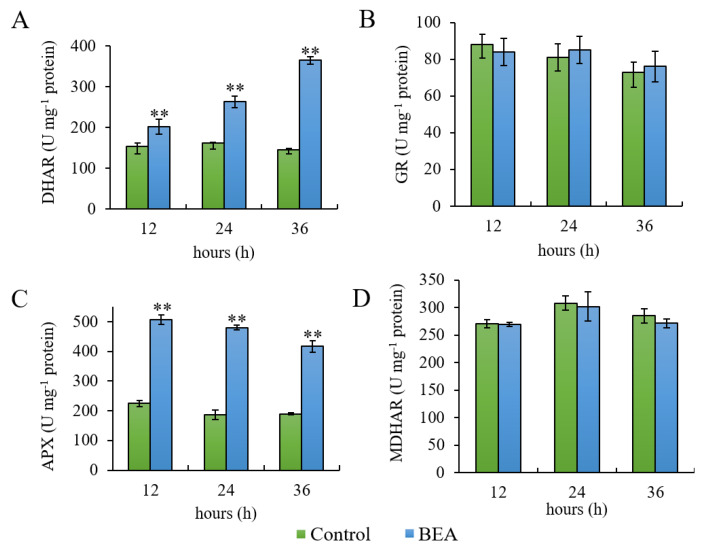
Enzyme activity of dehydroascorbate reductase—DHAR (**A**), glutathione reductase—GR (**B**), ascorbate peroxidase—APX (**C**) and monodehydroascorbate reductase—MDHAR (**D**) in control and beauvericin (BEA) treated samples during 36 h of assay. One unit (U) corresponds to 1 nmol of the substrate metabolized in 1 min. Asterisks indicate values significantly different from the control at each time point, according to the Student’s *t* test with *p* < 0.01 (**).

BEA treatment induced a significant increase in only two of the enzymes involved in ASC metabolism, namely DHAR and APX, while no difference was registered for MDHAR and GR (Figure 3). DHAR activity increase was time-dependent and statistically significant at all time points (*p* < 0.01) (Figure 3, Panel A). Likewise, APX increase was statistically significant at all time points (*p* < 0.01), with the highest increment being registered after 12 h (Figure 3, Panel C). GR (Figure 3, Panel B) and MDHAR (Figure 3, Panel D) levels remained constant and not statistically significant throughout the assay in both control and BEA treated samples.

### 3.3. Electrophoretic Analyses

**Figure 4:**
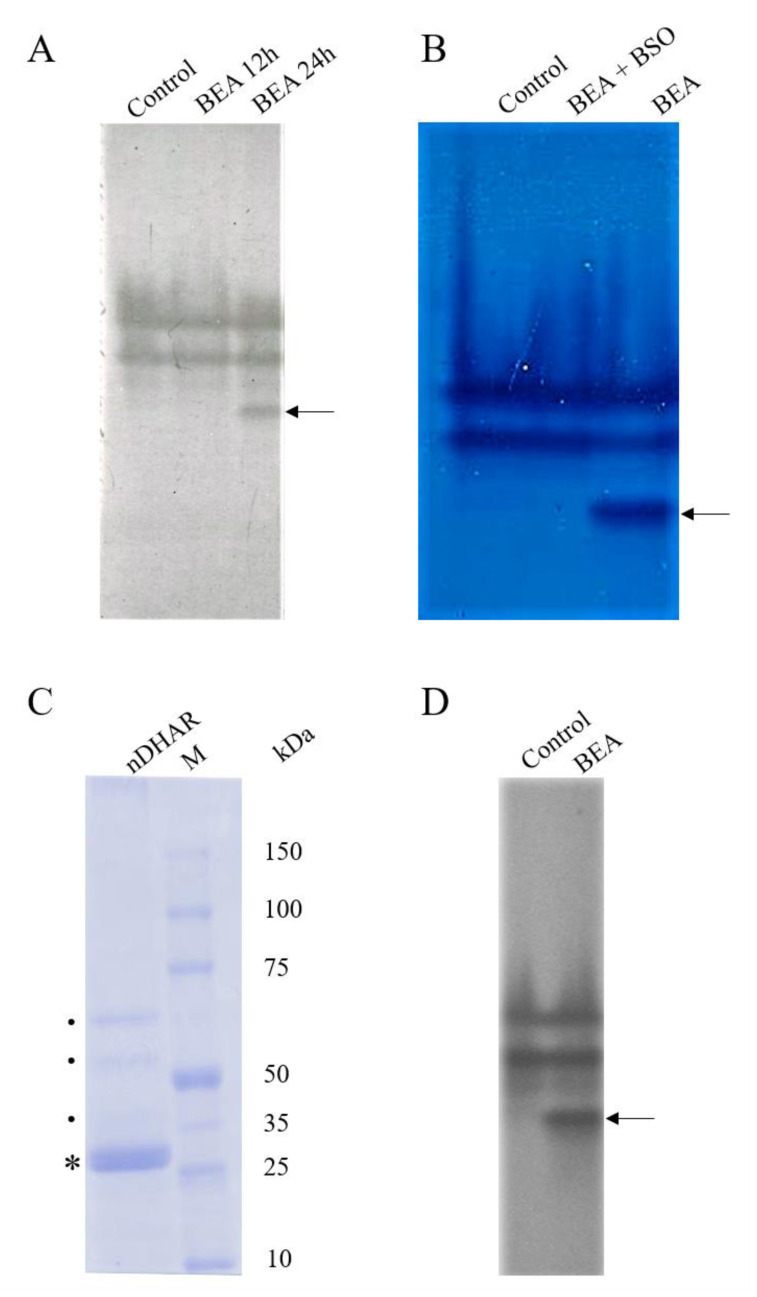
Electrophoretic analysis of DHAR proteins. (**A**) Native-PAGE of the cytosolic extract of the control and beauvericin (BEA) treated sample using DHA and GSH as substrates after 12 h and 24 h; (**B**) Native-PAGE of the cytosolic extract of the control, BEA and BEA plus BSO treated samples after 24 h; (**C**) SDS-PAGE of the novel band (nDHAR) eluted from the native-PAGE. The asterisk (*) in (Panel **C**) indicated the band subjected to mass spectrometry analysis. while the dots (•) indicated the other visible minor bands; (**D**) Western blot of the cytosolic fraction of the control sample and BEA; the experimental procedure is given in Section 2.7. Arrows in Panel A, B, and D indicate the novel DHAR. BEA = beauvericin; BSO = Buthionine-[*S*,*R*]-sulfoximine; M = Molecular weight marker.

To verify the time course of the band appearance, BEA treated samples were loaded on native-PAGE at 12 h and 24 h (Figure 4, Panel A). 

The electrophoretic pattern of the native-PAGE showed the occurrence of a new, additional band (indicated by the arrows in Figure 4, Panels A, B, and D) in BEA treated sample in comparison with the control when GSH was used as an electron donor. This band was detectable starting from 24 h of BEA treatment (Figure 4, Panel A) and did not appear when plants were incubated with BEA plus BSO, underlining the GSH-dependence (Figure 4, Panel B). 

These data were in accordance with the registered progressive increase in DHAR activity. No visible bands were detected when NADPH, alfa-lipoic acid or DL-lipoamide were used as electron donors alternatively to GSH (data not shown). The absence of any DHAR band on the light blue background (due to the non-enzymatic ASC reduction), once more suggested that all DHARs, including the novel, BEA-induced one, were GSH-dependent.

The new band was eluted from the native-PAGE and loaded on SDS-PAGE to estimate its molecular weight. As shown in Figure 4 (Panel C), the protein with DHAR activity appeared as a non-homogenous preparation because at least two more bands are visible in between 50 and 75 kDa, and potentially more in 35–50 kDa range. This suggested that the DHAR appearing as one band in the native-PAGE may be ascribable to either multiple proteins, or degradation of a larger protein, or both. 

### 3.4. Western Blot Analysis

To confirm that the protein under evaluation was a DHAR, the Western blot analysis of the cytosolic fraction was performed. All bands detected by the native-PAGE were recognized by the DHAR1 antibodies, thus confirming that this novel protein could be identified as a DHAR (Figure 4, Panel D).

### 3.5. Protein Identification by Mass Spectrometry

The mass spectrometry analysis was performed on the major band, the one displaying a molecular weight consistent with that of the classical DHARs (indicated by the asterisk in Figure 4, Panel C). Two different proteins were found in the same band, namely a putative chloroplast mRNA-binding protein and a chloroplast sedoheptulose-1,7-bisphosphatase (SBPase). The unique peptides and relative mass to charge ratio are reported in Table 1. However, both were reported to possess a molecular weight of 41–42 kDa, indicating that this result could be a contamination or protein degradation during sample processing or analysis. Indeed, only fragments from the C-terminal end of the proteins were detected by mass spectrometry (data not shown). 

### 3.6. Kinetic Measurements

**Figure 5:**
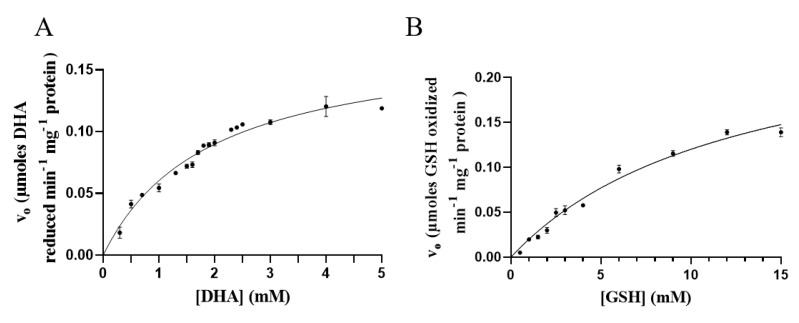
Michaelis–Menten plot of the rate of conversion of DHA (**A**) and GSH (**B**) by the novel DHAR.

Kinetic parameters of novel DHAR were determined for GSH and DHA. As reported in Figure 5, GSH and DHA reduction followed a Michaelis–Menten kinetic. *V*_max_ was 0.2731 μmol min^−1^ mg protein^−1^, *K*_M_ = 12.71 mM using GSH as substrate. As regards DHA, *V*_max_ was 0.1775 μmol min^−1^ mg protein^-1^ and *K*_M_ was 2.00 mM.

## 4. Discussion

**Figure 6:**
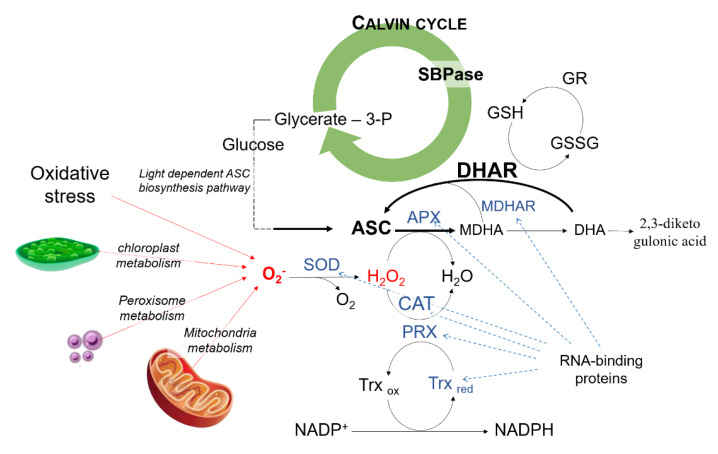
Plant antioxidant defense system. Reactive oxygen species (ROS) generated in the cell can be detoxified by the plant antioxidant system. The highly reactive and toxic superoxide ion (O_2_^−^) can be dismutated by superoxide dismutase (SOD) into O_2_ and H_2_O_2_. Catalases (CAT), peroxiredoxins (PRX), and peroxidases use H_2_O_2_ as a cofactor. Ascorbate peroxidase (APX) uses ascorbate (ASC) as an electron donor to detoxify H_2_O_2_ in a reaction that generates monodehydroascorbate (MDHA). MDHA can also undergo disproportionation into dehydroascorbate (DHA), which spontaneously decomposes to 2,3-diketogulonic acid. MDHA and DHA can be then restored to ASC by MDHA reductase (MDHAR) and DHA reductase (DHAR), respectively. The latter is a glutathione (GSH)-dependent enzyme and therefore uses GSH. Glutathione reductase (GR) restores the oxidized glutathione (GSSG) to its reduced form. Thioredoxins (Trx) are nicotinamide adenine dinucleotide phosphate^+^-dependent enzymes which regenerate PRXs. The possible involvement of the sedoheptulose-1,7-bisphosphatase (SBPase) and the RNA-binding proteins in the complex ascorbate regulation network is also presented in blue; more details are given in the discussion section.

Plants can counteract oxidative stress and, in general, biotic or abiotic stresses thanks to their antioxidants defense system such as ascorbate-glutathione pathways (Figure 6); the use of stress-resistant crops has been proposed as a new approach to increase the nutraceutical properties of vegetables and to understand how to promote the right pathways for bioactive compounds production [29]. 

The effects of BEA production by *Fusarium* spp in the field is still questioned. In fact, BEA is a mycotoxin able to induce oxidative stress, cytotoxicity, and cell death in vitro [10]. These activities are thought to be mediated by the ionophoric property of BEA, which causes cation entrance, particularly Ca^2+^, to the cells [5,30]. To date, little is known on the effect of BEA on plant crops. Tomato plants, for example, showed to counteract BEA induced oxidative stress [27]. Understanding the effect of BEA on plant crops is essential also to promote its use as an insecticidal and nematocidal molecule. In plant cells, cytoplasmic Ca^2+^ concentration increases in response to specific stimuli due to the activation of Ca^2+^-permeable channels. They can be both voltage-dependent and independent [31]. Ca^2+^ has several cellular targets, such as calmodulins and calmodulin-like proteins, Ca^2+^-dependent proteins, kinases, and calcineurin-B-like proteins [32]. Amongst them, Ca^2+^-dependent NADPH oxidases have recently been studied to link intracellular Ca^2+^ increase to ROS production [31]. Ca^2+^ cytosolic levels and ROS are involved in a self-amplifying loop. This mechanism is part of a complex and multifaceted network to balance the antioxidant systems and the ROS production and might explain to some extent BEA induced oxidative stress in plants.

Under our experimental conditions, ASC and GSH pools increased, together with DHAR and APX activities, most likely as a response to BEA-induced oxidative stress. Indeed, ASC is directly involved in radical scavenging (i.e., tocopheroxyl radicals, lipid peroxides, or oxidized metal ions) due to its ability to donate electrons and to contribute to H_2_O_2_ scavenging, serving as a cofactor for APX [18,33]. ASC pool is regulated by several pathways, including synthesis, recycling, degradation and transport. In our study an increase in DHAR, but not in MDHAR, the other ASC regenerating enzyme, was registered in BEA treated samples. This has been already reported as a common plant response to stress [26]. Additionally, in chloroplasts, the oxidized ASC can be alternatively reduced by ferredoxin-, glutathione-, and NAD(P)H-dependent pathways [14].

GSH is another pivotal molecule in plant metabolism, as it can be oxidized in several reactions linked to H_2_O_2_ detoxification [34]. In particular, GSH is the electron donor in DHA reduction, which is catalyzed by the DHAR. The increase in GSH pool with an unchanged GR activity could be probably due to an ex novo GSH biosynthesis rather than to the enzymatic reconversion of GSSG to GSH by GR. In addition, GSH metabolism is a robust and redundant metabolic system; redundant genes and different enzymes other than GR can modulate GSH pool [35]. The increase of the GSH pool is in accordance with the measured increase DHAR activity, and the expression of a novel, GSH-dependent DHAR, as demonstrated by the absence of DHAR activity after incubation with BSO. Indeed, three bands were visible in the native-PAGE of BEA treated samples and in tomato, only two different DHAR genes (*DHAR1* and *DHAR2*) have been identified [36]. Under stress condition, plants can express new DHAR isoforms, as reported for the alkaloid lycorine, which was able to induce the expression of novel proteins with DHAR activity in maize [37]. This can also be explained by the fact that DHARs are important enzymes that also participate in plant germination growth, development, and stress resistance [38,39]. 

DHARs are monomeric thiol enzymes, with a molecular mass of roughly 24 kDa. Similarly, the SDS-PAGE analysis revealed a major band having a molecular weight of roughly 26 kDa. For classical DHARs, apparent *K*_M_ for DHA ranges from 0.07 to 0.50 mM, whereas from 0.04 to 10 mM for GSH [14]. The apparent *K*_M_ of the novel DHAR was slightly higher than that of the classical DHARs, namely 2.00 mM for DHA and 12.71 mM for GSH. This suggests a lower level of activity, as reported for other chloroplast enzymes with a side DHAR activity [14]. In these proteins, the DHAR activity seems to be related to the presence of reactive cysteine residues in the active site [40], which are also present in the classical DHAR sequences [41]. Indeed, the overall amino acid sequence of those enzymes was distinct from that of DHARs already reported in literature [41].

The mass spectrometry analysis of the major band did not detect any DHAR-related peptides, at least in the molecular weight range of the classical DHARs. The major band was constituted by two different proteins—the SBPase and the RNA-binding protein. Those proteins have not been reported to possess DHA reducing activity yet. However, other proteins, such as trypsin-like proteins, thioredoxin reductases, glutaredoxins, protein disulfide isomerases, discolorins, and the 3-α-hydroxysteroid dehydrogenase were shown to possess also DHAR activity [14,42,43], meaning that those proteins can reduce DHA as a result of a “side” activity. Nonetheless, it cannot be excluded that the SBPase and the RNA-binding protein may be part of a protein complex that possesses DHA reducing activity or function as auxiliary proteins, together with the proteins which showed a higher molecular weight in the SDS-PAGE (Figure 4, Panel C).

SBPase is a key enzyme in the Calvin cycle, the primary pathway for carbon fixation in higher plants. It catalyzes the dephosphorylation of sedoheptulose-1,7-bisphosphate to sedoheptulose-7-phosphate; this reaction is crucial because at this point assimilated carbon can be used to regenerate ribulose 1,5-bisphosphate or to synthesize sucrose, starch, isoprenoid, or shikimic acid derivatives in the dark phase [44]. Therefore, it can be speculated that the novel DHAR may increase ASC pool by two different mechanisms, i.e. by reducing DHA, and increasing glucose pool for ASC biosynthesis. In this scenario, GSH pool may regulate the dual activity of the SBPase with DHAR activity (Figure 6).

SBPase is regulated by light, redox status, pH, and Mg^2+^ content [45]. SBPase was reported to possess at least three different cysteine residues, which may also be involved in its redox activation [46]. In particular, the use of a calcium ionophore was reported to lower stromal Mg^2+^ content, impair the Mg^2+^/H^+^ counter exchange, causing acidification and increasing SBPase activity [47]. Since BEA is a calcium ionophore [5,30], it can be speculated that SBPase can be also increased by BEA. Therefore, its effect on this particular enzyme deserves further investigations to better define the true identity of the DHA-reducing protein and its possible roles in the plant antioxidant system. SBPase has been reported to be a target to improve photosynthesis and plant resistance to biotic and abiotic stresses. In particular, in tomato, SBPase activity increase lead to a higher photosynthetic rate and efficiency, along with higher resistance to chilling-induced oxidative stress [48,49]. The current knowledge of SBPase is still limited, primarily due to the difficulty in purifying functional enzymes and in obtaining stable preparations of the enzyme [46]. Nevertheless, it may represent an important cell target for the improvement of tomato defense. 

The chloroplast mRNA-binding proteins are proteins involved in RNA processing. Their levels were reported to increase in tomato following wounding or cold storage [50,51]. Its functional role is not clearly understood. It may participate in the structural stabilization of RNA, or in regulating RNA export to enhance the expression of signaling pathways or genes involved in plant defense [52]. Interestingly, ferredoxins were reported to be capable of binding RNA with high affinity, and in a redox-dependent manner [53]. In *Arabidopsis*, several proteins involved in the plant response to cold, high salinity, osmotic stress, and heat, or which have a role in the intermediate metabolism, were reported to possess known RNA-binding domains [54]. This suggests that proteins may have a dual functional role, acting as enzymes and RNA-binding proteins (Figure 6) [54]. Based on the results presented, it cannot be excluded that also the novel DHARs may also be able to bind the RNA and exert regulatory functions. Indeed, amongst the enzymes involved in the ASC-GSH pathway, catalase, superoxide dismutase, APX, and MDHAR were already proposed as novel RNA-binding proteins candidates [54]. 

## 5. Conclusions

BEA is an emerging mycotoxin and its ecological role is still questioned. Tomato plants are able to counteract BEA induced oxidative stress. An important role may be ascribed to the induction of a novel protein with DHAR activity. The novel protein was able to reduce DHA to ASC only using GSH as electron donor, even if its affinity for these substrates was lower than classical DHAR already studied in plants. The mass spectrometry analysis did not detect any DHA-reducing related peptides at the molecular weight of classical DHARs but revealed a SPBase and a mRNA-binding protein. This novel BEA-induced DHAR adds a new pathway in the complex ascorbate regulation network, which may also involve protein with multifaceted activities, protein complexes, and auxiliary proteins, which contribute to DHA reduction during BEA induced oxidative stress.

Further studies will be needed to understand the mechanism through which BEA induces the novel protein to increase the ascorbate content and antioxidant potential of plants avoiding the use of transgenes and genetically modified organisms. Identifying all bands appearing in the native and SDS-PAGE, also in the presence of other stress inducers, will be needed to understand the nature of the novel protein with DHAR activity and its role. Increasing DHAR activity by means of BEA treatment would be helpful to increase the plant antioxidant defense system against biotic and abiotic stresses, the plant nutraceutical value, and the health benefits of consuming such products in the diet.

## Figures and Tables

**Table 1:** Protein assignment of the band cut from the SDS-PAGE.

Assigned Protein	Accession Number	Protein Coverage	Sequence	*m*/*z*
Chloroplast sedoheptulose-1,7-bisphosphatase	C5IU71	15.7%	HEFLLLDEGK	1669.80
			YTGGMVPDNQIIVK	1633.82
			YTGGMVPDNQIIVK (ox)	1633.82
			FEETLYGSSR	1188.57
			TTYVLALK	908.49
			MFSPGNLR	921.45
			GIFTNVTSPTAK	1236.67
chloroplast mRNA-binding protein	Q9XEJ6	14.9%	AVTLDGMAR	948.47
			IFNCVSDR	952.44
			FSEITGAGGR	993.49
			NMHFYAEPR	1163.52
			DCEEWFFDR	1254.48
			ILEGEVFDAVLDNNGK	1731.87
